# Remote Body Temperature Monitoring in Infectious Diseases: Pilot Studies in Different *Artiodactyla* Models

**DOI:** 10.3390/biology15191728

**Published:** 2026-10-01

**Authors:** Timm Konold, Sarah Wallworth, Christina Roccia, Jenny Jarman, Samirah A. Mannan, Pauline M. van Diemen, Helen E. Everett, Helen Crooke, Amin Asfor, Gareth J. Jones

**Affiliations:** 1 Department of Pathology & Animal Sciences, Animal & Plant Health Agency Weybridge, Addlestone KT15 3NB, UK; sarahwallworth@live.co.uk (S.W.); christina.roccia@gmail.com (C.R.); jenny.jarman@apha.gov.uk (J.J.); 2 Department of Virology, Animal & Plant Health Agency Weybridge, Addlestone KT15 3NB, UK; samirah.mannan@apha.gov.uk (S.A.M.); paulinem.vandiemen@apha.gov.uk (P.M.v.D.); helen.everett@apha.gov.uk (H.E.E.); helen.crooke@apha.gov.uk (H.C.); amin.asfor@apha.gov.uk (A.A.); 3 Department of Bacteriology, Animal and Plant Health Agency Weybridge, Addlestone KT15 3NB, UK; gareth.jones@apha.gov.uk

**Keywords:** body temperature, thermal microchip, cattle, sheep, alpaca, pestivirus, influenza virus, bovine tuberculosis

## Abstract

Thermal microchips are increasingly used in animals as an alternative to rectal temperature measurements because handling is not required. These pilot studies aimed to determine the usefulness of orally administered thermal microchips in infectious disease studies of *artiodactyla* species when a temperature increase was expected. Thermal microchips were administered subcutaneously to two groups of seven alpacas infected with influenza C and D virus respectively and three alpacas serving as non-challenged controls and orally to one alpaca in each group. Three sheep exposed to border disease virus and 12 cattle with experimental bovine tuberculosis when subjected to tuberculin skin testing were also given microchips orally. A temperature rise was observed in microchips and rectal temperatures around the same time in one alpaca and one sheep following infection, but was also recorded in orally administered microchips earlier during the night. A temperature rise was detected in all skin test-positive cattle except for one calf with a negative skin test and the least visible granulomatous lesions in the chest. Orally administered thermal microchips are a promising addition to rectal temperatures to determine body temperature changes in real time regardless of the time of day, to study physiological responses to infectious or immunogenic agents and possibly refine humane end-points in experimental studies. Factors to consider are bolus size, battery power and network connectivity to prevent data gaps (seen in alpaca and sheep) or excretion via feces (in sheep).

## Simple Summary

As an add-on to specific infectious disease studies, pilot studies were carried out in three species, three alpacas, three sheep and 12 cattle, exposed to infectious agents (influenza virus, border disease virus and *Mycobacterium bovis*) to assess whether thermal microchips given by mouth would be retained in the forestomach and record body temperature changes like rectal temperature monitoring, with the advantage that they can record temperature 24 h remotely. Body temperature rises were seen in one alpaca and one sheep coinciding with a rise in rectal temperature around the same time or even earlier at night, as well as evidence of infection. A temporary temperature rise was also detected in all cattle following tuberculin skin testing, although it was minimal in one animal that had a negative skin test, even though it had mild pathological lesions of tuberculosis. Data loss was encountered due to poor transmission to a recording device, loss of battery power or excretion of the microchip via the rectum (in two sheep), which needs to be considered in further studies. The microchips are a promising non-invasive tool to record body temperature to monitor changes over time, which may be useful to refine experimental protocols.

## 1. Introduction

Infectious disease studies in large animals, particularly if they involve experimental challenge, are tightly regulated to ensure best welfare for the animals under study. Infection with pathogens often produces pyrexia that is detectable by rectal temperature monitoring and is used to determine onset of disease and subsequently make decisions on further actions. However, this usually requires animals to be restrained to insert a rectal thermometer and personnel to handle the animals, which is time-consuming and thus usually only carried out once or twice a day at times convenient for the operator but not necessarily the time when pyrexia is first detectable. Handling of excitable animals may also increase rectal temperature [1], which is avoided when body temperature is measured remotely.

Various thermal microchips have been developed to monitor body temperature, which either used Radio Frequency Identification (RFID), which are smaller but require an operator with a transponder held near the animal to record the temperature, or are battery-operated to transmit temperature data continuously to a recording device or a cloud-based system without the need for an operator but are larger [2,3]. The latter is preferred as it records temperature continuously without the intervention of an operator and can be accessed in real time. The position of the thermal microchip will determine how closely the temperature resembles the rectal temperature, which is generally the “gold standard”. Implantation of the microchip in the periphery, e.g., under the skin, may be more prone to fluctuations due to environmental factors (lying on a cold floor, animals huddled together), which is prevented in abdominal implantation, but is more invasive; ideally, less invasive options are preferred.

*Artiodactyla* with different stomach compartments offer the advantage by which thermal microchips can be administered orally, e.g., as boluses with minimal if any side-effects and—if large and heavy enough, like rumen magnets or mineral-releasing boluses—are not excreted via the feces, thus enabling temperature monitoring over a longer period of time. Some of these products have already been available commercially to aid in monitoring temperature data, often in combination with accelerometers [4]. However, their use in infectious disease studies has been rarely documented.

The studies described here involved the exposure of *artiodactyla* (alpaca, sheep and cattle) to infectious agents (influenza virus, pestivirus and *Mycobacterium tuberculosis* respectively) for specific purposes. This provided the opportunity to run pilot studies to determine the usefulness of two orally administered thermal microchips (Moonsyst and anipill) to measure temperature changes following infection or immunological stimulation. It addressed the question of whether these boluses were practical (easy to administer and retained in the animal) and would detect changes similar to a rectal thermometer, including those that would be otherwise missed by conventional methods that required an operator. It was expected that the exposure to both viruses would cause pyrexia [5,6], particularly during the viremic phase, which is usually determined by measuring rectal temperature. Tuberculosis skin testing with purified protein derivates administered intradermally may cause a body temperature rise in infected cattle, as seen after subcutaneous (s.c.) injection up to 24 h after injection [7]. Experimental farm animal studies generally require the definition of humane end-points based on the display of specific clinical signs, which often include temperature measurements, but 24 h monitoring by an operator is not practical. If remote temperature monitoring was possible, comparable to rectal temperatures in terms of temperature changes, and data were accessible in real time, it could lead to a refinement in experimental studies.

## 2. Materials and Methods

All procedures involving animals were approved by the UK Home Office following ethical approval by APHA’s Animal Welfare and Ethical Review Body (license numbers PP7633638, PP9264766, PP7961670).

As the administration of a thermal microchip was supposed to be non-invasive and retention in the body was required, *artiodactyla* species were selected, which may retain an orally administered thermal microchip in a forestomach. The studies were not carried out solely to evaluate thermal microchips, and the species were selected based on the availability of studies that offered the opportunity to test the microchips. All animals were subjected to blood sampling under gentle manual restraint (sheep, alpacas) or restraint in a crush (cattle), but this was never done prior to rectal temperature measurements if performed.

Animals were either purchased from British farms for the purpose of the infectious studies (alpaca, cattle) or generated in-house (sheep). They were health-checked to confirm fitness for the study, and cattle and alpacas were derived from an officially tuberculosis-free region of Great Britain. Freedom from disease of interest was confirmed by serological tests in blood samples taken from the alpacas [5] and sheep (see below for tests) prior to their enrollment in the studies. Animals were housed on straw (and wood shavings for sheep) in accordance with the code of practice for the care and accommodation of animals bred, supplied or used for scientific purposes by the UK Home Office in secure biosafety level 2 (sheep, ambient temperature at winter avoiding excessively high temperatures) and temperature-controlled biosafety level 3 (set at 18 °C, alpacas, cattle) accommodation. They were fed pelleted concentrates once or twice daily in addition to hay and water provided ad libitum. Humane end-points were defined for each study (described in detail for alpacas [5]) based on clinical signs expected for the disease of interest (mainly respiratory signs and/or worsened general condition, which included rectal temperature in alpacas and sheep), and were monitored at least once daily.

### 2.1. Alpacas

The experimental infection of alpacas with influenza virus (family *Orthomyxoviridae*) to evaluate susceptibility and antibody responses has been described in detail elsewhere [5]. There were a total of 17 animals, which were 24–36 months of age and male. Briefly, two groups of seven alpacas were intranasally inoculated with either influenza C (ICV) or influenza D virus (IDV) and euthanized at 3 (three per group), 7 (two per group) and up to 20 (two per group) days post-inoculation (dpi) with pentobarbitone intravenously (Pentoject, Animalcare, Moorside, UK). A control group of three alpacas were mock-inoculated and euthanized three days later. All animals were monitored daily for signs of disease, which included measurement of body temperature with a rectal thermometer (Digi-temp Express, Kruuse, Langeslov, Denmark, or high-speed digital thermometer, Braun, Kronberg im Taunus, Germany) and a biothermal microchip (IDentiChip, Destron Fearing/Datamars, Lamone, Switzerland, subsequently referred to as chip) injected subcutaneously in the tissue above the superficial pectoral muscle in the axillar fossa. This RFID chip is activated by a scanner held near the animal. Measurements were made in the morning alongside clinical monitoring using a clinical score sheet and before any other procedures, such as nasal swabbing or blood sampling, were undertaken.

To continuously monitor body temperature, a thermal microchip pill, (anipill, BodyCAP, Hérouville-Saint-Clair, France, subsequently referred to as pill), was used, which is a 17.7 mm long capsule with a diameter of 8.9 mm weighing 1.7 g, and transmits data to a monitor (Anilogger, BodyCAP, Hérouville Saint-Clair, France) via radio frequency communication (433 MHz), which allows up to eight animals to be monitored. Sampling periods can be set to 1, 2, 5, 15 and 60 min, and the capsule can store up to 2000 data points without uploading to the monitor before they are overwritten. This battery-powered microchip is primarily designed for use in rodents with little body fat and muscle mass to ensure good communication with the monitor, which was not always achieved in alpacas due to the size of the alpaca and the location of the monitor near metal structures, which affected transmission so that the monitor was eventually placed higher off the ground (see Supplementary File S1 for data gaps). Variable sampling periods were used to temporarily increase the communication time between the microchip and monitor, although only 30 min intervals were used for data analysis.

The pills were implanted subcutaneously in the medial femoral area in all 17 alpacas: after anesthesia with 3 mg/kg tiletamine-zolazepam (Zoletil 100, Virbac, Carros, France) and 0.45 mg/kg xylazine (Rompun 2%, Elanco UK AH, Hook, UK) administered intramuscularly, the skin area was shaved and cleaned with chlorhexidine solution (Hibiscrub, Mölnlycke, Mölndal, Sweden), an 1 cm incision was made, the microchip was inserted, and the wound was stapled.

In addition, one alpaca in each group was administered the microchip pill orally using a plastic bottle filled with electrolyte solution (Lectade, Elanco UK AH, Hook, UK) to encourage swallowing. It was assumed that the small pills would remain in the alpacas based on a study in goat kids where the capsule was administered orally and remained in the animals for the study period of up to 25 days [8]. The pills were retrieved at necropsy.

To assess infection, nasal swabs collected daily from day 1 post-inoculation and every other day from day 8 post-inoculation were examined by Polymerase Chain Reaction (PCR) as described previously [5].

### 2.2. Sheep

To study the infection of sheep with border disease virus, a pestivirus of the family *Flaviviridae* [9], ten mule-crossbred, fleeced sheep were housed in a pen that shared the same airspace as a persistently, naturally infected calf. Sheep were assessed daily for clinical signs, which included measurement of rectal temperature by a rectal thermometer every morning from day 7 to 83 post-exposure; the exact time of measurement was not recorded. In parallel, three sheep were given a thermal microchip (anipill) via the oral route as described above for alpacas, with sampling frequency set as every 15 min, although this was increased temporarily in one animal to every minute to facilitate data transfer to the monitor, but only data that matched the time point of the other pills were considered for the analysis. Pills were administered 2 days after the sheep were moved to the building (D2 post-exposure). All sheep were castrated males, 7 months (two sheep) and 20 months (one sheep) of age respectively, and kept for 85 days post-exposure (dpe) before euthanasia with Secobarbital Sodium and Cinchocaine Hydrochloride (Somulose, Dechra, Hadnall, UK). Pills were retrieved at necropsy if present.

Details about this study and diagnostic test results [10] will be published separately in more detail. Briefly, viremia and serological response were determined from blood samples taken weekly. Viremia was determined via Reverse Transcription quantitative PCR (RT-qPCR) of weekly blood samples using previously published primers 106F, 179R and 190R, and probes P-162 and P-128 for the detection of BDV and BVDV [11]. Seroconversion was monitored from serum samples taken at the same time using a commercial BVDV/BDV ELISA (BVDV/MD/BDV p80 Protein Antibody Test Kit, IDEXX, Westbrook, ME, USA) following the manufacturer’s instructions. Results were read at 450 nm following the manufacturer’s interpretation: a Sample-To-Negative control ratio (S/N) ≤ 40% was considered positive.

### 2.3. Cattle

To generate a biobank of peripheral blood mononuclear cells for in vitro bioactivity assay validation, 12 male calves, aged 8–9 months, of various breeds (British Friesian, Aberdeen Angus and Fleckvieh crossbreed) were inoculated as previously described [12]. As the aim was purely to generate blood from infected animals, non-challenged controls were not included. Briefly, cattle were intrabronchially inoculated with 2 mL *Mycobacterium bovis* suspension (4373 CFU, AF2122/97 strain, APHA, Addlestone, UK) via endoscope under sedation with 0.05 mg/kg xylazine (Rompun 2%, Elanco UK AH, Hook, UK) administered intravenously. Cattle were tested for skin reactions by single intradermal comparative cervical tuberculin skin test (SICCT) [13] 55 days after inoculation using Bovine and Avian Purified Protein derivatives (PPD-B and PPD-A, Thermo Fisher [Prionics], Lelystad, The Netherlands) and four different batches of DST-F (Lionex, Braunschweig, Germany), a recombinant fusion protein consisting of the mycobacterial antigens ESAT-6, CFP-10 and Rv3615c [14] on the neck; reactions were measured 72 h after injection. For the purpose of this report, only reactions to PPD-A and B were taken into consideration: an increased skin thickness difference of more than 4 mm in the PPD-B injection site compared to PPD-A after 72 h post injection was considered positive. A skin thickness difference below 4 mm at PPD-B compared to PPD-A was interpreted as inconclusive if there was at least 2 mm difference at the PPD-B injection site [13]. One day before skin testing, all cattle were administered a thermal microchip bolus (Moonsyst, Kinsale, Ireland) [15] via bolus applicator (Bovivet mini introducer, Kruuse, Langeskov, Denmark), followed by oral administration of electrolyte solution (Lectade, Elanco UK AH, Hook, UK) in a plastic bottle to ensure that the bolus was deposited in the reticulum. The bolus used was a small rumen bolus (75 mm length and 22 mm diameter) that measures temperature every 10 min. Data were uploaded to a cloud-based system using a gateway with a mobile SIM card. Cattle were housed in groups of four in biosafety level 3 accommodation, with the gateway located in the plant room above the pens. Rectal temperature measurements were not carried out because a possible temporary temperature rise would have been expected late at night or early in the morning. All cattle were euthanized by captive bolt and subsequent exsanguination the week after skin testing. Boluses were collected post mortem. Visible lesions in the lung and draining lymph nodes (granulomas) were assessed at gross necropsy as an indicator of infection with *Mycobacterium bovis* using a scoring system from 1 to 5 depending on size and distribution as described elsewhere [14].

### 2.4. Data Analysis

Statistical software GraphPad Prism GraphPad (version 8.4.2 for Windows, GraphPad Software) was used to determine normal distribution of data (Kolmogorov–Smirnov test) and carry out basic statistics. Comparison between different body measurement methods (rectal temperature and thermal microchips) was limited to alpaca and sheep and excluded specific statistical analysis due to the small number of subjects and the presence of missing data. Therefore, the temperature data from the three alpacas and sheep, respectively, which were measured by oral microchip in addition to rectal thermometer, were visualized by measurement method in Excel (Excel for Microsoft 365, Microsoft) and included the mean and difference between rectal temperature and oral pill values. Missing data were excluded from the analysis. For alpacas, temperature data were compared between the different methods taken at around the same time in the morning. As the exact time of measurement of rectal temperature in sheep was not known but fell between 07:30 and 10:30, and bolus temperature could vary by up to 0.7 °C during that time, the hourly pill data during this time period (up to four records, taken at approximately 07:30, 08:30, 09:30 and 10:30) were averaged for comparison with the rectal temperature.

To determine body temperature changes in cattle after administration of the skin test reagents intradermally, the baseline temperature was determined from each animal (=experimental unit) as the average of six records taken at 10 min intervals on the day of skin testing before 08:30 a.m. for comparison with the peak temperature within the 72 h of skin testing.

Temperature data were analyzed without knowledge of the infection status, although they were known to be exposed, with the exception of the alpacas where the control group was known from the outset.

## 3. Results

### 3.1. Alpacas

Basic values from the measurements by the four methods taken at around the same time for comparison are summarized in Table 1. The highest median and mean were recorded with a rectal thermometer, followed by oral pill and chip, and the s.c. temperature had the lowest median and mean.

The temperature data in the three animals that were obtained from all four measurement methods (rectal thermometer, chip, s.c. and oral pill) are visualized in Figure 1; the mean and difference between rectal and oral pill temperature are also displayed.

The means of all rectal and oral pill temperature differences were 1.2 °C (variance 0.12) in alpaca 1880, 0.6 °C (variance 0.22) in alpaca 2019 and 0.8 °C (variance 0.33) in alpaca 1953. Basically, temperatures measured by rectal thermometer were on average higher than temperatures measured by oral pills.

Table 2 lists the values obtained by the pills collected every 30 min over the same time period separated by group and individual alpaca. As described previously [5], viral RNA indicative of viremia, when pyrexia may be expected, was detected in nasal swabs from ICV and IDV-inoculated alpacas at D1–8 for ICV and at D1–7 for IDV and peaked between days 2–6 for both viruses, but alpaca 2019, which was considered febrile at D12 with clinical signs later warranting euthanasia, was not viremic at that time anymore. Its temperature peak was on D12 at night (see Table 2), although temperatures above 40 °C were also seen at D14 in the morning (see Figure 1B).

### 3.2. Sheep

Table 3 provides data collected from the sheep given the oral pill. Data collection for each sheep was not uniform throughout the study because of loss of the pill or battery power, which meant that not all the data were transferred to the monitor. This was seen towards the end of the battery life of the pill where transmission became more erratic. Temperature peaks detected by each method were not at similar time points, but rectal temperature was only measured once daily in the morning.

Rectal temperature and pill temperature, as well as the mean and difference between rectal and pill temperature in the three sheep, are displayed in Figure 2.

The means of all rectal and pill temperature differences were −0.3 °C (variance 0.14) in sheep 7501, −0.4 °C (variance 0.20) in sheep 7614 and −0.3 °C (variance 0.15) in sheep 7618. Contrary to alpacas, temperatures measured by the pill were on average higher than temperatures measured by rectal thermometer.

A temporary temperature peak at or above 40 °C was detected by both methods suggestive of pyrexia at 63 dpe in sheep 7618, although the pill temperature reached its maximum above 41 °C in this sheep at 57–58 dpe (see Table 3).

Viremia based on RT-qPCR was not present before 78 dpe in 7501 and seroconversion was not detected during the monitoring period. Viremia was detected at 42 dpe in 7614 (seroconversion at 57 dpe) and 64 dpe in 7618 (seroconversion at 78 dpe). Whilst pill temperature data were not available for these days in 7501 and 7614, the raised temperature in 7618 at around the same time suggested a link with viremia.

### 3.3. Cattle

Baseline temperature prior to tuberculin skin testing was 38.6–39.6 °C. Depending on the individual animal, the maximum temperature measured ranged from 39.4 to 42.0 °C, which was first observed between 8 and 14 h post injection. Table 4 shows the results for individual cattle.

The majority of cattle had a positive skin test result (SICCT ≥ 4 mm) and a body temperature difference exceeding 2 °C, with the exception of two cattle: one was skin test-inconclusive despite a body temperature difference exceeding 2 °C and one had only a minor temperature rise of less than 1 °C, and the skin test was negative. All body temperature increases occurred at least 8 h after the skin test injections and returned to baseline the following morning, except for two cattle (5660, 5661), when this happened the following afternoon. An example of the temperature changes is given in Figure 3.

On gross necropsy, all cattle had visible lesions suggestive of infection, although the lesion score was the lowest in 5667, with lesions confined to lungs and not lung lymph nodes, followed by 5665 and 5669. The highest lesion score was recorded for animals 5661, followed by 5658 and 5664.

## 4. Discussion

This study evaluated the use of thermal microchips in different *artiodactyla* species as a potential substitute for rectal temperature measurements if retained in the forestomach, which is less invasive than parenteral administration. The use of microchips that enable continuous recording over time without interference with the animal was preferred because it enables recordings of temperatures at unsociable times, when temperature rises may otherwise be missed. If febrile conditions occur after experimental challenge of animals with pathogens that only cause a temporary temperature spike, it may be erroneously interpreted as subclinical infection and may also give misleading information on incubation times if pyrexia is the predominant indicator of infection. Continuous temperature monitoring is also useful if it is part of an experimental protocol where clinical score sheets are used to determine humane end-points and one of the clinical signs is body temperature. It may lead to refinement (one of the 3Rs [16]) to increase clinical sign monitoring when a temperature rise is first detected.

As the studies had considerable limitations, which are discussed further below, they can be considered primarily as pilot studies to demonstrate that orally administered boluses can be used to record body temperatures and detect changes in temperature that would be missed by conventional rectal temperature monitoring once or twice daily. These studies were not specifically designed to validate oral thermal microchips against rectal temperature measurements used as the gold standard; they provided merely the opportunity to trial orally administered thermal microchips to continuously monitor body temperature and study their retention in the animal. As such, sample size was small, bolus use was dictated by availability, and staffing for other procedures relevant to the studies (e.g., sampling of blood) made it challenging to follow a study design that would have been more appropriate if thermal microchip evaluation was the sole purpose.

Two statistical tests are usually used to compare different methods. The correlation coefficient informs on the relationship between two variables, but not necessarily on agreement, whereas the Bland–Altman plot is used to describe agreement between two quantitative measurements by plotting every difference between two paired methods against the average of the measurements [17]. Whilst the latter can be modified for repeated measurements and data that are not normally distributed, which was the case with our data even after transformation (square or cube root transformation of data), a purely descriptive analysis with graphical display of the difference between rectal and oral chip temperature and their mean was preferred due to the limited number of animals and data that could be compared. It demonstrated that rectal temperature was higher than oral pill temperature in alpacas and the difference could be more than 1 °C. In sheep, the rectal temperature tended to be below pill temperature and the difference was mostly varying around −0.5 °C. The fluctuation in the difference between rectal and pill temperature mostly matched changes in rectal temperature. The data suggested that the baseline using the oral pill is clearer with less noise compared to rectal temperature. In the few cases where a temperature increase suggestive of pyrexia was recorded, the oral pill detected the same event as the rectal temperature measurement, alpaca 2019 at D13–14 (see Figure 1B) and sheep 7618 at D63–67 (see Figure 2C), even though the pill data was averaged in sheep as the exact timing of rectal temperature measurement was not recorded.

The correlation of subcutaneously implanted thermal microchips with rectal temperature has been studied in multiple animal models, including sheep [18] and cattle [19,20], with rectal temperature generally being higher. Correlation may be adequate in the species studied, which is encouraging, but the temperature difference means that a reference range has to be established for subcutaneously implanted thermal microchips, often using a baseline reading prior to infection. By contrast, body temperature in the forestomach is more comparable to the rectal temperature [21], which was confirmed in our study, since the temperatures were similar to those measured by a rectal thermometer and described as reference range in the literature (alpaca: 35.1–39.4 °C [22], sheep (38.3–39.9) and cattle 38.0–39.3 °C [23]). There was a deviation from the baseline above 2 °C in the cattle (see Table 4), which resulted in temperatures of 41 °C or above. Both baseline and increased temperature would have also been interpreted as normal and pyrexia if measured with a rectal thermometer.

As the thermal microchips are located in the forestomach, temperature drops occur, for example, when the animal ingests cold food or water [21,24,25,26], which was also experienced here (see Figure 3). It is not known why rectal temperatures were on average higher in alpacas but lower in sheep, but this was based on a small sample size. Different fermentation processes because of anatomical differences or different roughage digestibility in alpacas [27] may have caused the difference.

The temperature differences seen in subcutaneously implanted versus injected thermal microchips in alpacas were likely caused by the different anatomical regions, which have been observed in other species [19]. The implantation in the axillary region may be preferable to the medial femoral region because temperatures were closer to rectal temperatures. The intercostal region was used in a study in Guanacos, but rectal temperatures were not measured for comparison [28].

The temperatures measured with the microchips were not validated for correctness prior to the study, although they had a certificate issued by the manufacturer and measured room temperature uniformly when not in use. Whilst the thermometers were regularly conformance-checked, and staff followed a standard operating procedure to measure rectal temperature, there is a possibility that rectal temperature measurements may not always be accurate as they are more prone to operator variability than microchips. Studies in ruminants and pigs have shown that measurements can be influenced by the procedure itself, type of thermometer, and the penetration depth into the rectum [23,29,30,31]. This may have contributed to the larger variability in rectal temperatures in sheep and alpacas compared to the anipill data, despite always being measured in the morning to account for circadian variability.

It is known that ambient temperatures can affect body temperature, although it was not considered to be relevant in the present studies. Both alpacas and cattle were housed in temperature-controlled buildings that did not deviate from the thermoneutral zone (0 to 25 °C in cattle [32], −10 to 20 °C in the closely related guanaco [33]). Sheep were housed during winter, thus avoiding excessive high ambient temperatures. It has been shown that colder ambient temperatures generate higher amplitudes of daily oscillation of rectal temperature in sheep, although those were still within the normal range [34]. In general, there were daily variations in body temperature, with the highest peak usually in the afternoon or evening, but none were 40 °C or above [22,35].

Whilst the application of thermal microchip boluses is a considerable improvement to monitor body temperature remotely and its application in infectious disease research is in its infancy, there were some issues that need to be considered. Boluses for cattle are commercially available, particularly for dairy cows where they can be used to detect estrus, but require an adequate internet connection to upload the data, which can be an issue if connectivity is poor. The system here relied on a mobile phone network, which was often poor in the building where the cattle were housed and needed to be reset frequently. For alpacas and sheep, a microchip was used that was designed for smaller species and thus much smaller, with less battery power and, consequently, range to connect to a monitor within the pen to upload the data, even though the monitor was retrospectively fitted with an antenna to improve the range. This led to data losses as the microchip could only hold a maximum of 2000 data points. Suitability was based on a study in goats where this microchip was used successfully, but it struggled with alpacas, which sometimes required the operator to hold the monitor next to the abdomen to upload missing data. The system did not require an internet connection (data could be uploaded from the monitor to a computer at a later stage), so it is preferable for animals housed in pens where internet connectivity is poor or absent. Data loss was experienced in sheep due to loss of battery power in the microchip (these were reused, but the lifespan of the battery could not be foreseen with the battery power indicator) and loss via excretion. A study in sheep led to the recommendation to use boluses of small diameter (e.g., <15 mm), which the bolus fulfilled, but it was too light for a recommended weight greater than 20 g [36]. Other researchers used data loggers in the rumen, but the size and weight were not specified [34,37]. The same bolus was retained in alpacas, but the monitoring method was considerably shorter, and it cannot be excluded that it would have been excreted eventually.

Skin testing for bovine tuberculosis (bTB) diagnosis resulted in an increase in body temperature in the majority of cattle. This was based on thermal microchips only without rectal temperature measurements, although a rise in rectal temperature has been used historically to diagnose bTB after s.c. injection of tuberculin [7]. It is unlikely that environmental factors contributed to the temperature rise. Rumen temperature was reported to rise after feeding concentrates [21] but this effect is temporary, not expected to occur at night, and the rise was considerably less than seen in the current study. The body temperature increase appeared to agree with the interpretation that a temperature increase of 2 °F, equivalent to 1.1 °C, after s.c. injection of tuberculin (not intradermal as used here) would be considered positive, whilst an increase of less than 1 °F, equivalent to 0.6 °C, would be classified as negative [7]. The only animal (5667) that had the least temperature increase and was skin test-negative despite the presence of minimal visible lesions would have been interpreted as doubtful, with a temperature of 103 °F, equivalent to 39.4 °C, after injection [7]. The timing of the increase would make this impractical for a routine test if reproduced with a conventional rectal thermometer. However, it is expected that farmers will make more use of novel remote monitoring technologies in the future, and temperature monitoring after tuberculosis skin testing may be a useful addition to measurement of skin thickness, which could be explored in future studies. Whilst temperature changes were observed after intradermal injection of 0.1 mL rather than subcutaneous injection of a comparatively higher volume of up to 3 mL [7], it is possible that the simultaneous injection of six skin test reagents intradermally had a more pronounced effect on body temperature than the injection of bovine and avian tuberculin only. There was no opportunity to test infected animals with only these two reagents for comparison.

In alpacas, the observed rise in body temperature was not necessarily linked to viral shedding. Viral shedding in nasal swabs peaked between 2 and 6 dpi for both viruses [5], but this was not reflected in a temperature rise in all animals at around that time. Temperature rises of similar magnitude were also seen in non-challenged alpacas. The only considerable rise above 40 °C was seen in alpaca 2019 at 12–13 dpi for several hours at night (see Table 2) when viral shedding was supposed to have stopped [5]. This oddity, which led to euthanasia of the alpaca, may have been the result of immunosuppression caused by influenza virus and subsequently superinfection.

Infection of sheep with pestivirus happened late after exposure (earliest 42 dpe), which was unfortunate because of the loss of data early on in two sheep (7501, 7614), which was before viremia occurred. Rectal temperatures were considered to be within the normal range although pill temperature was 40.4 °C at 19 dpe (see Table 3). In cattle, rectal temperature was reportedly lower than temperature measured in the rumen/reticulum because of heat produced by fermentation [25], and the pill temperature of 40.4 °C in this sheep may thus be normal. Sheep 7618 had a rectal and pill temperature of 40 °C and above at 63 dpe (see Figure 2C), suggestive of pyrexia, which coincided with viremia detected at 64 dpe. Blood samples were collected weekly, and viremia was not present at 57 dpe. There were intermittent temperature peaks in the pill above 41 °C suggestive of pyrexia starting at 13:14 on 57 dpe, but whether this is linked to viremia and could predict when blood samples should be taken remains speculative and requires more data.

## 5. Conclusions

Despite limitations in terms of data from the studied population, the preliminary results suggest that orally administered thermal microchips that record temperature remotely are promising tools to monitor temperature changes in *artiodactyla* species, following exposure to infectious agents where the microchip remains in the forestomach for considerable time. Thermal microchips may need to be developed specifically for different species and different environments to prevent data loss via fecal excretion or because of poor communication between microchips and the reader.

## Figures and Tables

**Figure 1:**
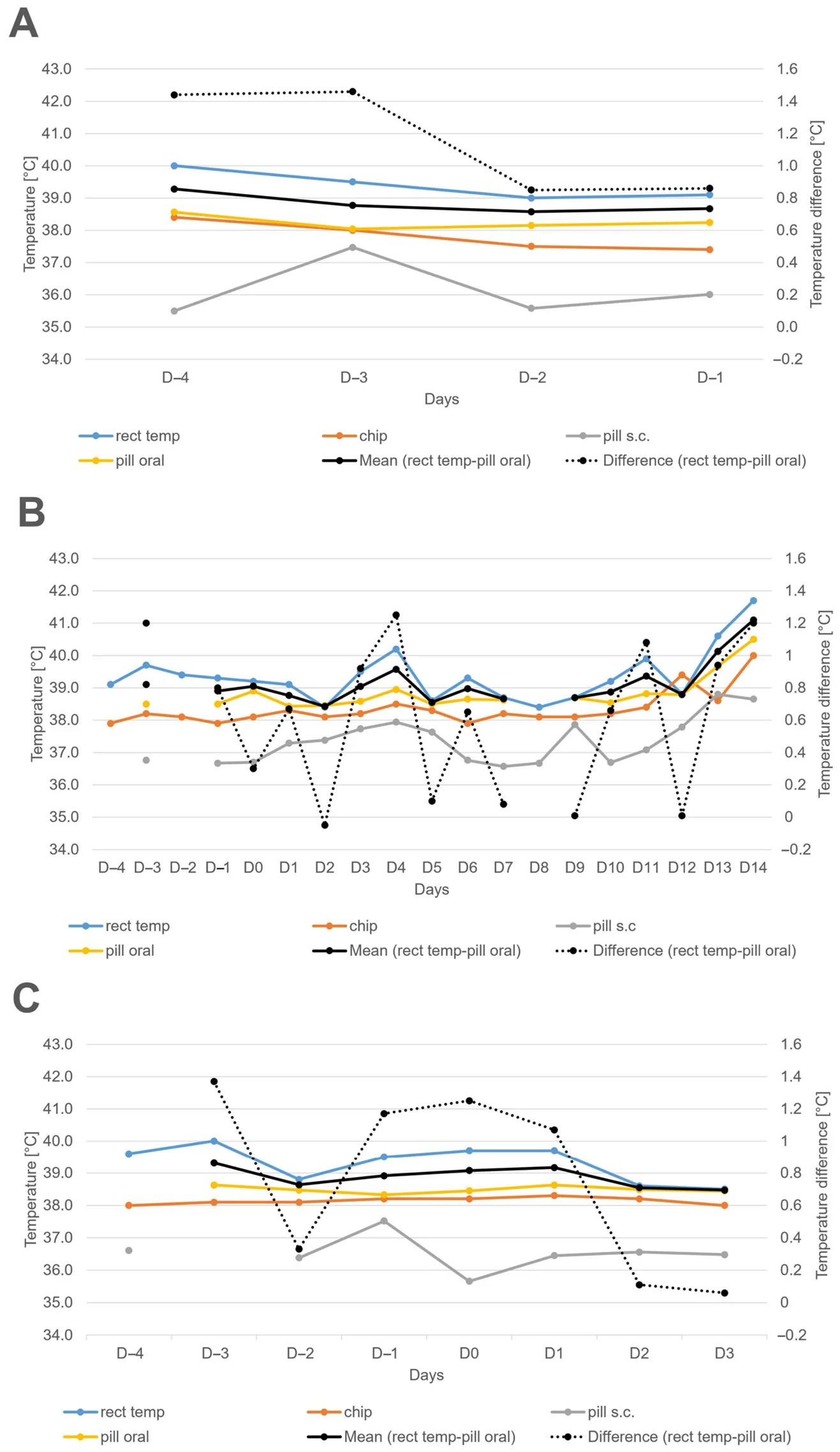
Temperature graphs of the three alpacas with all four measurement methods around the same time of the day. (**A**) Control alpaca 1980, (**B**) alpaca 2019 challenged with influenza C virus, and (**C**) alpaca 1953 challenged with influenza D virus. Missing data points were due to instances when contact with the monitor could not be established temporarily. Pill s.c. is the subcutaneously inserted pill; rec temp = rectal temperature. The *y*-axis on the right of the graph refers to the temperature difference between the rectum and oral pill (dotted black line in the graph); for example, the temperature difference is close to 0 °C on day 3 in alpaca 1953 (**C**).

**Figure 2:**
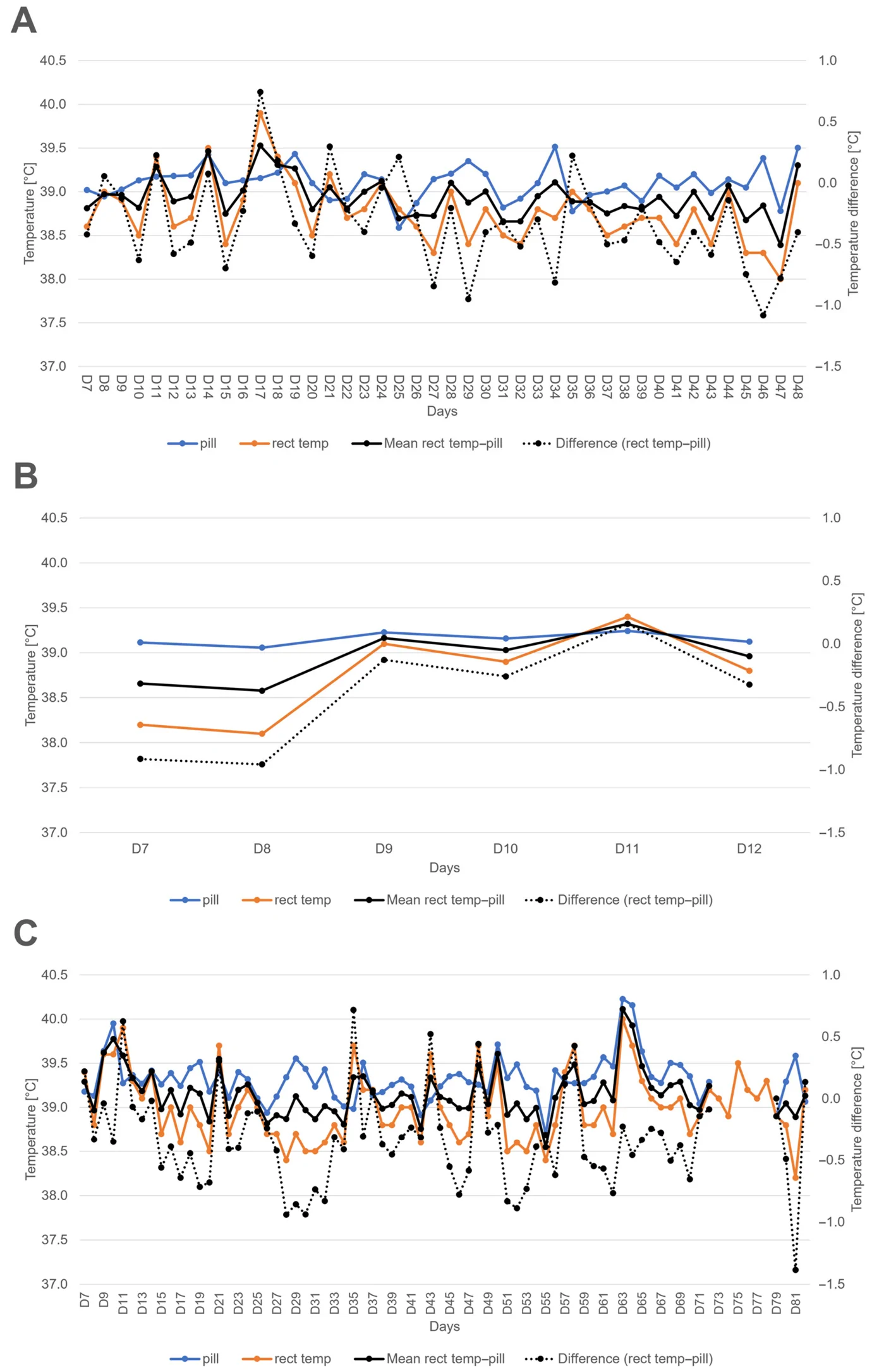
Rectal temperature compared to pill temperature around the same time in the morning. (**A**) Sheep 7501, (**B**) sheep 7614, and (**C**) sheep 7618. Pill data were averaged to cover the morning period when rectal temperatures were measured because the exact timing was not recorded. rec temp = rectal temperature. The *y*-axis on the right of the graph refers to the temperature difference between the rectum and oral pill (dotted black line in the graph); for example, the temperature difference is less than 0.5 °C on day 12 in sheep 7614 (**B**).

**Figure 3:**
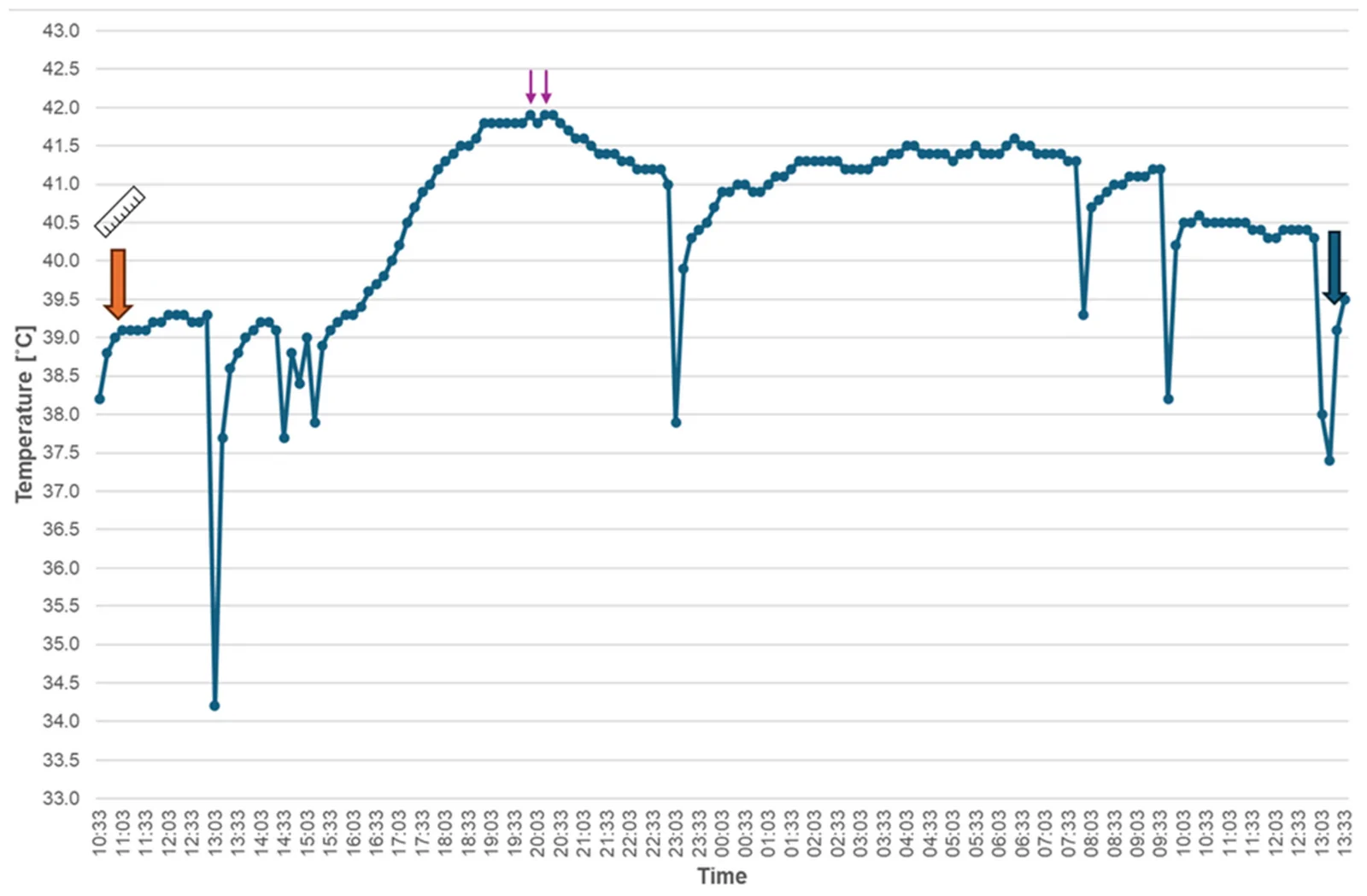
Body temperature of calf 5661 recorded every 10 min on the day of skin test until return to baseline the following day. The orange arrow with ruler indicates the time of skin test, the maximum was reached twice in the evening (small purple arrows) and the temperature returned to baseline the next afternoon (blue arrow). Negative temperature spikes were likely caused by the calf drinking, which were ignored to estimate time to return to baseline temperature.

**Table 1:** Median, mean, range and standard error of mean of measurements.

Method	*n*	Median [°C]	Mean (Range); SEM [°C]
Rectal temperature	194	38.8	38.8 (37.8, 41.7); 0.04
Oral pill	27 *	38.5	38.7 (38.0, 40.5); 0.09
Chip	194	37.9	37.9 (36.9, 40.0); 0.03
Subcutaneous pill	179 *	36.5	36.6 (34.5, 38.8); 0.05

*n* is the number of temperature data; SEM is the standard error of mean. * Some pill temperatures were not transmitted to the monitor: 15 from the subcutaneous pill and four from the oral pill.

**Table 2:** Descriptive statistics and time of highest temperature of pill measurements taken every 30 min from individual alpacas.

Group	Alpaca	*n*	Median [°C]	Mean (Range); SEM [°C]	Time Point of Maximum Temperature
Control	1998	166	37.2	37.2 (35.1, 38.1); 0.04	D−4: 19:32, 20:02; D−3: 01:32
Control	1980	168	37.4	37.1 (31.8, 38.4); 0.08	D−4: 18:32
Control	2025	164	37.7	37.3 (33.7, 39.3); 0.10	D−4: 02:32
Control	1980 OR	167	38.4	38.4 (36.5, 38.9); 0.02	D−2: 21:03
ICV	1967	310	36.2	36.2 (32.6, 37.8); 0.04	D−3: 23:33
ICV	1993	1063	36.7	36.7 (33.6, 38.1); 0.02	D19: 20:02
ICV	2028	426	36.8	36.9 (34.7, 38.3); 0.03	D3: 23:31
ICV	1923	356	36.9	36.9 (35.5, 38.5); 0.03	D3: 09:32
ICV	2010	420	36.9	36.9 (34.8, 37.9); 0.03	D2: 18:33; D3: 02:32
ICV	2019	796	37.1	37.3 (35.8, 40.4); 0.03	D12: 22:02
ICV	1917	302	37.4	37.4 (36.1, 39.1); 0.03	D1: 17:34
ICV	2019 OR	702	38.8	38.9 (37.1, 41.0); 0.02	D12 *: 22:32
IDV	1864	329	36.5	36.6 (34.9, 37.9); 0.03	D−3: 20:04
IDV	1983	538	36.7	36.6 (34.2, 37.9); 0.03	D−2: 19:32
IDV	1961	533	36.8	36.8 (34.5, 38.2); 0.02	D6: 19:32
IDV	1953	336	36.8	36.9 (35.1, 38.7); 0.04	D−3: 20:34
IDV	2003	1173	37.0	36.9 (34.5, 38.5); 0.02	D−4: 21:01
IDV	2017	1141	37.1	37.1 (34.6, 39.1); 0.02	D4: 01:00
IDV	1871	363	37.8	37.8 (35.7, 39.1); 0.04	D−3: 21:34
IDV	1953 OR	318	38.7	38.6 (36.6, 39.3); 0.02	D3: 00:01

*n* is the number of temperature data; SEM is the standard error of mean. OR refers to the animals with oral pills. ICV = influenza C virus, IDV = influenza D virus, and D = Day. * Temperature over 40.0 °C between D12: 19:32 and D13: 02:01.

**Table 3:** Age, descriptive statistics and time of highest temperature from the three individual sheep.

Sheep	Age [m]	Method	*n*	Median [°C]	Mean (Range); SEM [°C]	Time Point of Maximum Temperature
7501 ^a^	20	P	4094	39.1	39.1 (32.4, 40.4); 0.005	19 dpe: 15:43
		RT	77	38.6	38.6 (38.0, 39.9); 0.04	17 dpe: a.m.
7614 ^b^	7	P	978	39.3	39.3 (31.9, 39.9); 0.016	6 dpe: 18:07
		RT	77	38.7	38.7 (38.0, 39.9); 0.04	19 dpe: a.m.
7618 ^c^	7	P	6525	39.3	39.4 (33.7, 41.6); 0.006	57 dpe: 15:00 *
		RT	77	39.0	39.0 (38.2, 40.0); 0.04	63 dpe: a.m.

m = months; *n* = number of temperature data; SEM = standard error of mean; P = Pill; RT = Rectal Temperature; dpe = days post-exposure; and a.m. = ante meridiem (before midday). ^a^ Lost contact after 48 dpe, excreted via feces and lost; 350 (9.6%) of 3653 data points missing until then. ^b^ Lost contact after 13 dpe; excreted via faeces and lost; 41 (7.7%) of 535 data points missing until then. ^c^ Retrieved at necropsy (85 dpe) but lost battery power at 82 dpe; 1197 (19.7%) of 6084 data points missing until then. * Temperature above 41 °C between 13:14 on 57 dpe and 00:46 on 58 dpe.

**Table 4:** Temperature data from the 12 different cattle inoculated with *Mycobacterium bovis*.

Animal	Baseline [°C]	T_max_ [°C] (Time [h:min] After Injection)	∆T [°C]	Duration of T_max_ [min]	Comment	SICCT [mm]
5665	39.6	42.0 (09:08)	2.4	30	biphasic T_max_	2
5661	39.1	41.9 (08:59)	2.8	40	biphasic T_max_	33
5663	39.4	41.8 (10:31)	2.4	100	biphasic T_max_	25
5662	39.6	41.7 (11:34)	2.1	20		22
5668	39.0	41.7 (11:49)	2.7	20		14
5659	38.9	41.6 (11:53)	2.7	30		12
5658	39.3	41.5 (13:37)	2.2	80	biphasic T_max_	22
5666	39.0	41.3 (08:13)	2.3	260	triphasic T_max_	15
5669	39.0	41.3 (13:02)	2.3	60	biphasic T_max_	16
5660	38.9	41.2 (10:16)	2.3	70	biphasic T_max_	19
5664	38.8	41.0 (13:01)	2.2	120	biphasic T_max_	38
5667	38.6	39.4 (13:59)	0.8	80	biphasic T_max_	0

Baseline as determined from six recordings prior to skin test injection. ∆T refers to the difference between baseline and maximum temperature. T_max_ is the maximum temperature recorded in 24 h format and its duration in minutes. Single intradermal comparative tuberculin test (SICCT) result is measured in millimeters. Some animals had multiple maximum temperature peaks (two, biphasic or three, triphasic), thus prolonging the duration of the maximum temperature.

## Data Availability

The original contributions presented in this study are included in the article/Supplementary Materials. Further inquiries can be directed to the corresponding author.

## Supplementary Materials

The following supporting information can be downloaded at: https://www.mdpi.com/article/10.3390/biology15191728/s1, Supplementary File S1. Temperature data.

## Abbreviations

The following abbreviations are used in this manuscript:

bTB	Bovine tuberculosis
dpe	Days post-exposure
ICV	influenza C virus
IDV	influenza D virus
OR	Oral pill administered
P	Pill
PCR	Polymerase Chain Reaction
PPD-A	Avian Purified Protein derivatives
PPD-B	Bovine Purified Protein derivatives
RFID	Radio Frequency Identification
RT	Rectal temperature
RT-qPCR	Reverse Transcription quantitative PCR
s.c.	subcutaneous
SEM	Standard Error of Mean
SICCT	single intradermal comparative cervical tuberculin skin test
S/N	Sample-To-Negative control ratio
T_max_	Maximum temperature
